# Vari Morph Cast Iron—A High IQ Material—Structure, Properties, Ultrasonic Control, Technology and Industrial Application

**DOI:** 10.3390/ma19061212

**Published:** 2026-03-19

**Authors:** Jerzy Stanisław Zych, Marcin Myszka, Janusz Postuła, Sylwia Kobyłecka

**Affiliations:** 1 Faculty of Foundry Engineering, AGH University of Krakow, Aleje Mickiewicza 30, 30-059 Krakow, Poland; 2 FANSULD Cast Iron Foundry, ul. Zielona 22, 26-700 Końskie, Poland

**Keywords:** cast iron, graphite shape, ultrasonic measurements, properties, physical, mechanical, functional

## Abstract

Cast iron, whose structure simultaneously contains graphite precipitates in various forms, with controlled proportions of individual forms, has been named “Vari-Morph” (VM) cast iron by the authors. The authors have been researching the properties of such cast iron for many years, and the results are being published successively. This new type of cast iron, not included in national (Polish) or European standards, is intended as a material for special-purpose castings. These castings have unique requirements for a set of properties: physical, mechanical, and functional. VM cast iron is characterized by a set of properties that cannot be achieved when the graphite is uniform in shape. The desired properties of VM cast iron are achieved by controlling the morphology of graphite precipitates and the proportion of individual forms in the structure, rather than by changing the matrix. To quantitatively describe graphite precipitates, a proprietary method for determining the graphite shape indicator (f_K_) was developed. Graphite precipitate analysis is performed by scanning a microscopic image of the metallographic specimen, and then using Tescan Imaging Software (Tescan ESSENCE™) Unified Control for Imaging and Analysis, each precipitate is described using surface metrology parameters. The final value of the graphite shape indicator (f_K_) is calculated as a weighted average of all precipitates present in the analysis field. Empirical relationships between the f_K_ indicator and a selected group of physical, mechanical, and functional properties of VM cast iron were determined. Studies have demonstrated a very well-correlated relationship between the f_K_ indicator in VM cast iron and ultrasonic wave velocity (C_L_). The relationship C_L_ = f(f_k_) is characterized by a very high correlation coefficient of R > 0.90. In previous publications, the authors presented the relationships between the f_K_ indicator and physical properties such as thermal conductivity (λ), specific density (ρ), strength (R_m_), elongation (A5), index quality (IQ), and functional properties such as low-cycle mechanical fatigue resistance (Z_c_), thermal fatigue resistance (N), and cast iron tightness (H) as functions of the f_K_ index. The study concerned VM cast iron with a ferritic matrix. This work contains new empirical relationships that extend previous studies. The newly developed relationships replace the f_k_ shape indicator with the velocity of the ultrasonic wave determined in cast iron with a specific f_K_ indicator value. This resulted in a number of practical dependencies, including: λ = f(C_L_); ρ = f(C_L_); E_D_ = f(C_L_); R_m_ = f(C_L_); A5 = f(C_L_); IQ = f(C_L_); N = f(C_L_); Z_c_ = f(C_L_); H = f(C_L_). These relationships allow us to measure the wave velocity in a Vari Morph iron casting (with various forms of graphite) and determine a number of characteristics and properties of the material/iron from which the casting was made. It is possible to assess the suitability of a casting with a specific structure for operation under selected conditions.

## 1. Introduction

Cast iron containing carbon in the form of graphite, known as gray iron, is the most commonly used material in global foundry production. Cast iron for castings, based on the form (morphology) of graphite precipitates, is produced in three groups (PN EN 1560): cast iron with flake graphite (from EN GJL-100 to EN GJL-350), cast iron with vermicular graphite (from EN GJV-300 to EN GJV-500), and cast iron with spheroidal graphite (from EN GJS-350-22 to EN GJS-900-2). Cast iron and castings are not produced with intentionally mixed forms of graphite. Only a very small percentage of graphite other than the intended form is permissible, e.g., up to 5.0% in ductile iron and up to 20% in vermicular iron. To achieve such uniform graphite structures, the production technologies for individual grades of cast iron have been adapted. In many cases, limiting and maintaining a uniform graphite morphology (flake (L), vermicular (V), or spheroidal (S)) significantly limits the ability to freely shape many of the cast iron’s properties, both necessary and beneficial for casting applications. The form of graphite precipitates influences all cast iron properties and characteristics, including physical, mechanical, technological, functional, and operational properties. This limitation currently has no substantive justification.

**Figure 1:**
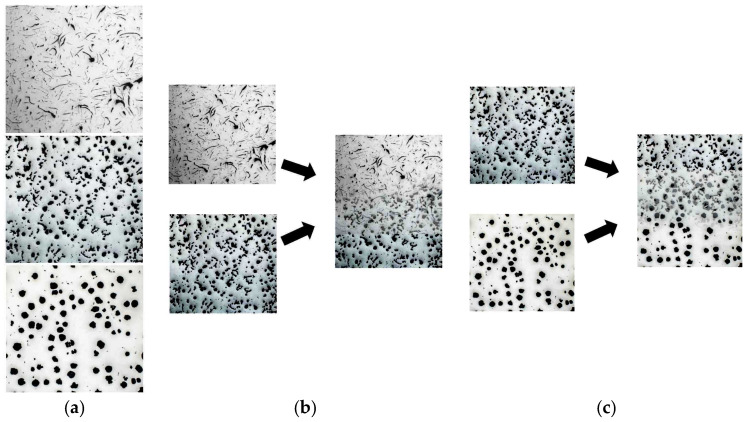
Types of graphite morphology: (**a**) flake, vermicular and spheroidal graphite; (**b**) cast iron with mixed forms of flake and vermicular; (**c**) cast iron with mixed forms of vermicular and spheroidal graphite.

The research conducted [1,2,3,4,5,6,7,8,9,10,11,12] indicates the possibility of producing cast iron with mixed graphite precipitate morphology and confirmed the possibility of controlling this morphology in a deliberate and predictable manner. Figure 1 presents the concept of cast iron with mixed graphite form, which has been given the name Vari Morph (VM) [1]. The EN ISO 945 standard specifies only three types of graphite form in raw castings that have not been heat treated [13,14]. The remaining types, crossed out in Figure 1a, cover the graphite precipitate forms in cast iron after heat treatment. VM cast iron encompasses both the group of graphite precipitate forms shown in Figure 1b,c. The idea of VM cast iron is a smooth transition of the graphite form from flake, through mixed: flake + vermicular and mixed vermicular + nodular, to fully nodular. This smooth transition in the form of graphite precipitates will also enable smooth shaping of a whole range of physical and mechanical properties. Within the range of standardized cast iron grades, property changes are gradual.

The research described in this article demonstrated the significant influence of graphite precipitate morphology on the entire group of cast iron properties and confirmed the possibility of controlling these properties. One of the most important results that can be achieved is the ability to produce gray cast iron characterized by relatively good strength (R_m_—300–400 MPa) and simultaneously low hardness (HB—160–180). In traditional modified cast iron with homogeneous flake graphite (from EN GJL-250 to EN GJL-350), to achieve the described strength, the metal matrix is transformed into a purely pearlitic form, leading to an increase in hardness to HB—230–240. Modern casting machining methods using high-speed CNC machines are adapted to the processing of materials with reduced hardness. In the case of cast iron castings, hardness is expected to be maintained below 200 HB. If only the flake graphite form is retained, achieving these parameters (Rm and HB) is very difficult, and for higher Rm values, it is impossible. At hardnesses significantly above 200 HB, it is necessary to reduce the machining speed parameters of CNC machines, which is not conducive to economics. This was confirmed directly by research conducted as part of the project [8] at an iron foundry that uses CNC machining of its own castings. Cast iron quality is often measured by an index called Index Quality (IQ), which is defined as: IQ = R_m_/HB [15]. Gray cast iron has a relatively low IQ value of 1.0–1.4, while ductile iron ranges from 2.5–3.0. By controlling the graphite morphology and allowing the possibility of mixed forms in the casting structure, it is possible to continuously fill the IQ range and obtain values from 1.0 to 2.5. Vermicular cast iron partially falls between gray and ductile iron. However, the requirement of the European standard PN-EN 16079 (from EN-GJV-300 to EN GJV-500) to achieve 80% of vermicular graphite complicates the production technology on the one hand, and on the other limits the freedom in shaping many features of cast iron, including achieving a better IQ index and a number of other properties.

The production of cast iron in a form other than flake requires secondary treatment of the liquid metal, to which small amounts of additives (inoculants, cast iron spheroidizing mortars) are introduced. The possibility of controlling the graphite morphology over a wide range of changes has been confirmed, among others, in the works of the authors [1,2,3,4,5,6,7,8,9,10,11,12,16,17,18] and many others. The work concerned increasing the resistance of cast iron to thermal fatigue by controlling the morphology of graphite precipitates over a wide range, from flake to nodular [9,10,11]. Furthermore, as analytical work conducted so far has shown, cast iron in which the graphite is not homogeneous in terms of its morphology is characterized by above-standard properties that cannot be achieved with a uniform graphite form [12]. Examples of VM cast iron structures correlated with f_K_ shape indicator are shown in Table 1.

Studies on the influence of the graphite shape index f_K_ (or ξ index) on the resistance of cast iron to thermal fatigue also show that there are strict relationships that link other features and properties of cast iron with the shape index ξ, such as: R_m_ = f(ξ), A5 = f(ξ), E_D_ = f(ξ), α (vibration damping) = f(ξ), λ (thermal conductivity) = f(ξ); Index Quality IQ = f(ξ) [5,6]. The shape index is defined in many ways. For example, the ξ index is defined as the area of the surface occupied on a metallographic microsection by the graphite separation to its circumference when squared. The authors propose defining the graphite shape indicator “f_K_” for VM cast iron as a weighted average of the values for the flake, vermicular, and spheroidal precipitate forms, taking into account the contribution of each form to the overall set of precipitates visible on the metallographic specimen. Because most cast iron properties are determined as a function of the graphite shape indicator, correctly determining its value is crucial in describing the structure of Vari Morph (VM) cast iron.

## 2. VM Manufacturing Technology—Method Selection

**Figure 2:**
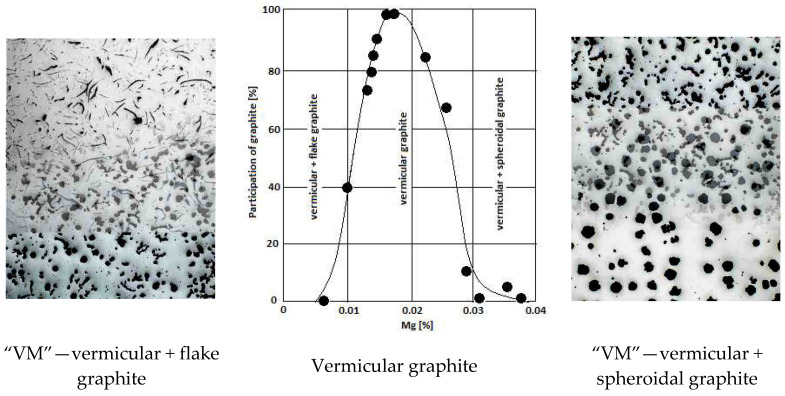
Morphology of graphite precipitation in Vari Morph cast iron.

Technological standards do not provide for the production of mixed graphite cast iron. In 2018, the authors [1] named mixed graphite cast iron with controlled proportions of individual shapes “Vari-Morph” (VM). Cast iron with the proposed graphite precipitate structure could become an interesting material for castings with special applications and requirements for the combination of physical and mechanical properties. Since that year, the team has continued work on the technology, structure, and properties of this type of cast iron [1,2,3,4,5,6,7,8,9,10,11]. VM cast iron is characterized by a set of physical and mechanical properties that cannot be obtained when the graphite precipitates are uniform in shape. VM cast iron falls between cast iron with flake graphite (F) and vermicular graphite (V), and between cast iron with vermicular graphite (V) and spheroidal graphite (S). Figure 2 presents the idea of the existence of a mixed form of graphite in the structure of cast iron as a criterion for qualification to the group of Vari Morph cast iron grades.

Cast irons with a L + V graphite mixture are characterized by good thermal conductivity and better plasticity and strength than gray cast iron. They can be used for structures and components subjected to rapid, cyclic heating, e.g., as a material for metal molds, slag ladles, ingot molds, etc. Of particular interest is cast iron with a mixed form of graphite: graphite (S) + graphite (V), with a high proportion of vermicular graphite. Such cast iron is characterized by high strength, good plasticity, and tightness. Furthermore, it has relatively good vibration damping properties and relatively good thermal conductivity, which are not found in ductile irons. In [1,2,3,4,5], it was demonstrated that such cast iron also has high resistance to thermal fatigue—higher than vermicular cast iron.

The authors’ research work [1,2,3,4,5,6,7,8,9,10,11,12] conducted both on a laboratory and industrial scale indicates the technical possibility of producing VM cast iron with mixed graphite morphology and confirmed the technological ability to control graphite morphology in a deliberate and predictable manner as to the obtained assumed structure of graphite precipitates.

One of the most important results achieved by producing Vari Morph cast iron is the ability to produce gray cast iron characterized by relatively good strength (R_m_—300–400 MPa) and simultaneously low hardness (HB—160–180). The graphite in such cast iron occurs in mixed, flake, and vermicular (F+V) forms. This cast iron has a ferritic or ferritic-pearlitic matrix. Traditional cast iron with only flake graphite (gray) has an almost purely pearlitic structure to achieve the described strength, reduced carbon content (low CE), and a hardness in the HB range of 230–240. Therefore, it has a reduced quality index (IQ), which in the case of cast iron is defined as the ratio of R_m_ strength to hardness (IQ = R_m_/HB). Modern casting machining methods using high-speed CNC machines are designed to process materials with reduced and uniform hardness. In the case of cast iron, hardness below 200 HB is often required. If the graphite flake form is maintained, achieving these parameters (R_m_ and HB) for cast iron with a strength above 250 MPa is virtually impossible. Vari Morph cast iron offers this possibility and opportunity.

Producing any type of cast iron other than flake graphite requires secondary metallurgy of the metal/cast iron in its liquid state. Small amounts of master alloys/additives (cast iron spheroidizing mortars and inoculants) must be added. Several secondary metallurgy methods are known in industrial practice to modify the form of graphite precipitates and eliminate flake graphite from the structure. This group of methods includes:

- Magnesium addition (controlled amount)
- Simultaneous addition of magnesium and titanium
- Addition of “Michmetal” alloy.
- Simultaneous addition of magnesium, cerium, aluminum, and calcium (Mg-Ce-Al-Ca).
- PQ-CGI Inmold process
- Method using Sinter-Cast technology
- Elkem method (COMPACTMAG magnesium-cerium mortar).

**Figure 3:**
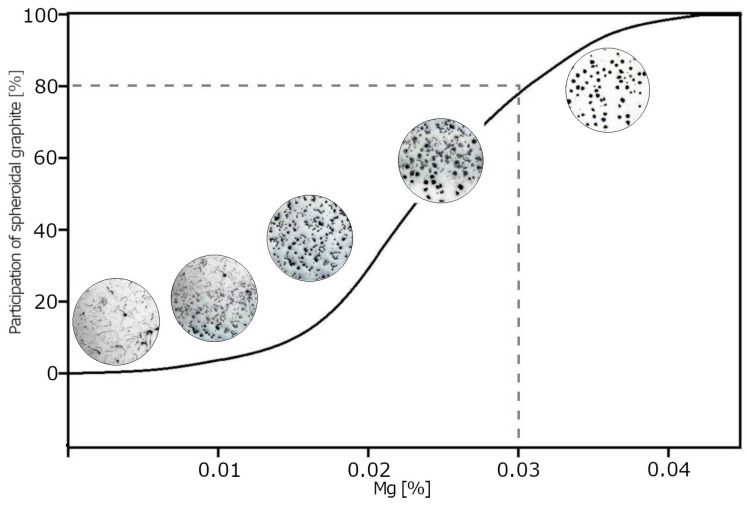
The influence of Mg content in cast iron on the share of nodular graphite in the structure.

As a result of many preliminary and verification tests conducted by the authors on the application of the above methods, it was concluded that the method of introducing a controlled amount of magnesium (Mg) into the molten metal (Figure 2 and Figure 3) is the easiest technology. Furthermore, it is characterized by high repeatability of obtained results and simplicity of application. To date, this method has been widely used in the production of ductile and vermicular cast iron. With precisely defined (residual) magnesium contents remaining in the solidifying casting, mixed forms of graphite can be obtained in Vari Morph cast iron, as shown in Figure 2 and Figure 3. Magnesium is introduced into the molten metal in the form of a low-magnesium FeSiMg mortar. The amount/content of magnesium introduced from the magnesium mortar into the cast iron and remaining as residual in the solidifying casting relative to the total amount of magnesium used in the spheroidization process is defined as the degree of magnesium assimilation. Its value ranges widely, from approximately 30% to over 80%. It depends on many technological factors, parameters, and the spheroidization technology/method. The authors tested three methods: the Tundish method using a tight-lid ladle, the PE method, and the in-mold spheroidization method—the Inmold process. The highest repeatability is achieved with the Inmold method, which allows for the precise and repeatable introduction of the planned amount of Mg into the cast iron. Using this method, it is possible to produce serial Vari Morph iron castings with any desired graphite precipitate morphology.

Quantitative description of graphite precipitates in the metallographic structure of cast iron, when individual precipitates of different shapes occur side by side, requires the use of mathematical statistics. The shape of a single precipitate is described by a shape index, the degree to which the shape approximates a circular cross-section. The shape index “f” is most often defined as the ratio of the surface area of the precipitate visible on the metallographic micrograph to the area of the circle described by that precipitate. The value of the “f” index defined in this way falls within the following ranges:

➢ f = 0.00–0.34  for flake graphite,
➢ f = 0.35–0.64  for vermicular graphite,
➢ f = 0.65–1.00  for spheroidal graphite.

In their works, the authors [1,2,3,5] introduced a new concept of “averaged shape indicator of graphite precipitates” (f_K_). In general, f_K_ is the weighted average of the shape indices of “all” graphite precipitates in the observed field of the metallographic cross-section. The methodology for determining the value of the f_K_ index is described later in the work.

To describe the effect of the morphology of graphite precipitates in VM cast iron on its broadly understood quality, empirical relationships were determined, in which the independent variable is the value of the average shape indicator of graphite precipitates f_K_ in connection with: mechanical properties (R_m_ = f(f_K_); A5 = f(f_K_); IQ = f(f_K_))); physical properties (C_L_ = f(f_K_); λ = f(f_K_); ρ = f(f_K_); and functional properties (N = f(f_K_), Zc = f(f_K_), cast iron tightness H = f(f_K_)). In the light of the research results described below, the empirical relationships are relatively well correlated. This provides the basis for the thesis that a number of properties can be predicted based on the value of the average shape index f_K_ of VM cast iron. This is particularly important if the f_K_ indices are determined directly in the casting.

## 3. Research on the Structure of VM Cast Iron—Determination of the Graphite Shape Indicator

The main goal of research on VM cast iron is to determine the relationships between the broadly understood shape of graphite precipitates in the cast iron structure and its mechanical, physical, functional, and other properties. Therefore, developing a methodology for determining the graphite shape indicator is crucial. Defining the graphite shape indicator for a single precipitate is relatively easy. Several methods for determining it can be found in the relevant literature. Table 2 provides the mathematical definition of the shape indicator in three variants and the range of changes in its value as it transitions from flake-like to mixed (F + V), vermicular (V), mixed (V + S), and finally spheroidal.

**Figure 4:**
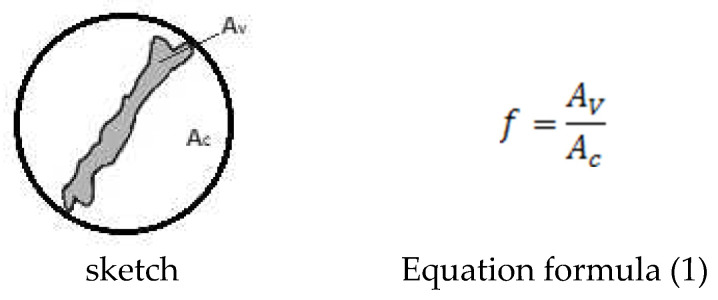
Scheme of shapes of graphite precipitates cross-sections—sketch and formula of defining graphite shape indicator f.

All variants of the shape indicator calculations use the relations between the parameters of the circle described on the graphite precipitate and its actual surface, as shown in Figure 4.

The graphite shape indicator f was determined for each precipitate, acc. to [19], in accordance with the equation (Figure 4 Equation (1)), where:

*A_v_*—surface of the graphite precipitate [mm^2^],

*A_c_*—area of the circle of a diameter equal to the highest particle dimension [mm^2^].


(1) $$f=\frac{A_{v}}{A_{c}}$$

It is important to remember that all attempts to describe the three-dimensional structure of cast iron, particularly the description of graphite precipitates based on a flat image of a metallographic microscope, are always a gross simplification. Table 3 compares an SEM image taken at a fracture of a cast iron sample with an optical microscope image obtained from a metallographic microscope. The images represent various forms of graphite.

To illustrate this degree of simplification, Table 3 presents fracture images obtained using SEM of three types of cast iron, alongside the appearance of the unetched metallographic microscope. In accordance with standards [13,14], the classification and structure description is based on the analysis of metallographic microscope sections using classical optical microscopy. For this reason, but with an awareness of the significant simplification, this work also uses the analysis of a flat image, which, with a plane, intersects the three-dimensional internal structure of the cast iron, as seen in the examples in Table 2.

It is rare for graphite precipitates of uniform shape to occur in the cast iron structure, particularly in grades containing vermicular or nodular graphite. In such a situation, to characterize the cast iron in terms of the shape of the graphite precipitates, an average value of the graphite shape factor must be determined. In this study, the average graphite shape indicator “f_K_” was determined by calculating its value as a weighted average. This means that the precipitates observed in the microsections were counted in individual shape groups and size classes. Then, after assigning them a value of the “f” factor, the weighted average shape factor was calculated.

**Figure 5:**
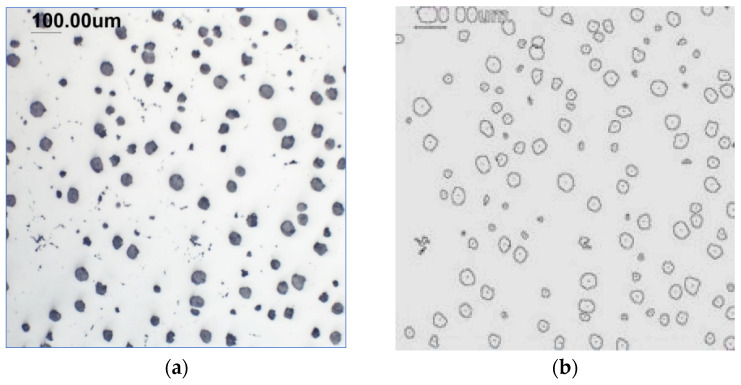
(**a**) Microstructure—optical microscope. (**b**) Precipitates with a diameter describing graphite d > 0.0785 µm.

Counting and describing all graphite precipitates in the traditional manner is extremely labor-intensive, even in a simplified version. Therefore, computer software for image analysis and statistical data processing was used. The assessment of graphite precipitate morphology was performed with the support of the ImageJ program ver.1,52a. A new procedure was developed for determining the average value of the graphite shape indicator (“f_K_”) [2]. The starting point is a metallographic image of cast iron without etching and its electronic recording, shown in Figure 5a. The image is then processed using ImageJ, which provides its recording, as shown in Figure 5b. Particles with sizes d < 0.0785 µm for flake and vermicular graphite and d < 0.010 µm for spherical precipitates were software-rejected. Not all black dots on a metallographic specimen represent graphite precipitates; very small black areas represent micro-voids located at eutectic grain boundaries. Therefore, they were rejected and omitted from further calculations of the graphite shape indicator. ImageJ allows for the analysis of many image element parameters, including graphite precipitates in cast iron. Table 4 summarizes those used to describe and determine the graphite shape index value. As an example, they refer to the image shown in Figure 5b.

**Figure 6:**
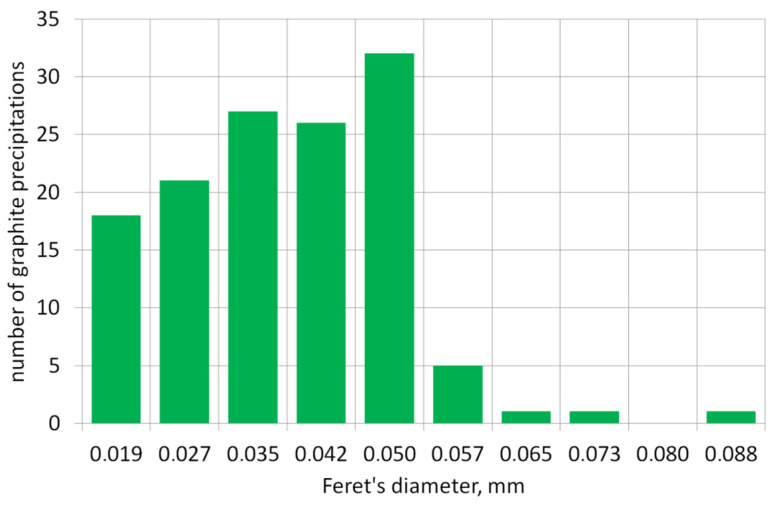
Number of graphite precipitates in Feret diameter size classes.

**Figure 7:**
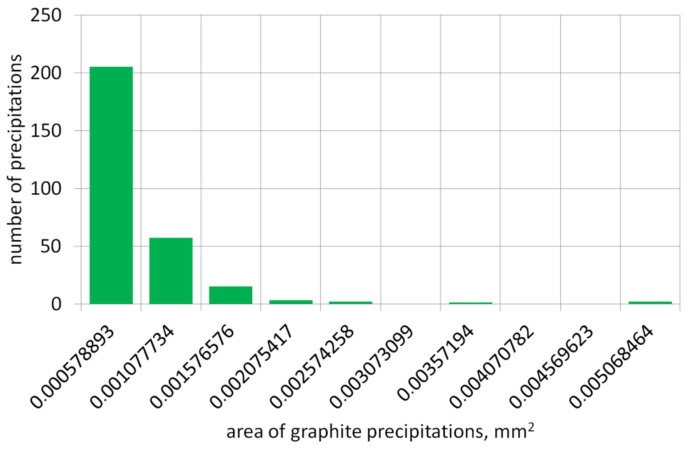
Number of graphite precipitates in surface size classes.

The graph below presents the results of the graphite precipitate analysis per mm^2^ of the tested surface/sample. Figure 6 shows the number of precipitates and their distribution across diameter classes, and Figure 7 shows the distribution of precipitates across surface size classes of individual graphite precipitates.

The further procedure for calculating the average value of the shape indicator f_K_ is performed in an Excel spreadsheet, based on the parameters obtained from ImageJ. For each precipitate, the surface area of the circle circumscribing the graphite precipitate was calculated based on its main dimension. The graphite shape indicator was determined from Equation (2).

(2) $$f=\frac{A_{v}}{A_{c}}$$

where:

*A_v_*—surface area of the precipitate,

*A_c_*—surface area of a circle with a diameter equal to the largest dimension of the particle.

The “f” indicator is a number whose theoretical value varies from slightly above 0 to 1.0, but in practice ranges from 0.20 to 0.90. In the literature, characteristic values for uniform graphite morphology of each type of graphite are assumed [20]:

- 0.00–0.34—flake graphite
- 0.35–0.64—vermicular graphite
- 0.65–1.00—nodular graphite

For Vari Morph cast iron, in the methodology adopted by the authors for calculating the average value of the shape indicator f_K_, graphite precipitates are grouped into three classes based on their shape, as shown in Table 5. Column 2 provides the number of graphite precipitates in each class, and column 3 provides the percentage share of the total number. Column 4 gives the average (arithmetic) values of graphite shape indices for each shape class calculated from Equation (3). Column 5 gives the average value, which is calculated as a weighted average, according to Equation (3). Equation (3) calculates the sum of the products of the values from column 3 multiplied by the value from column 4 and converted into a dimensionless value.

(3) $$f_{K}=\frac{\Sigma (participation\;\%)\cdot f}{100}$$

**Figure 8:**
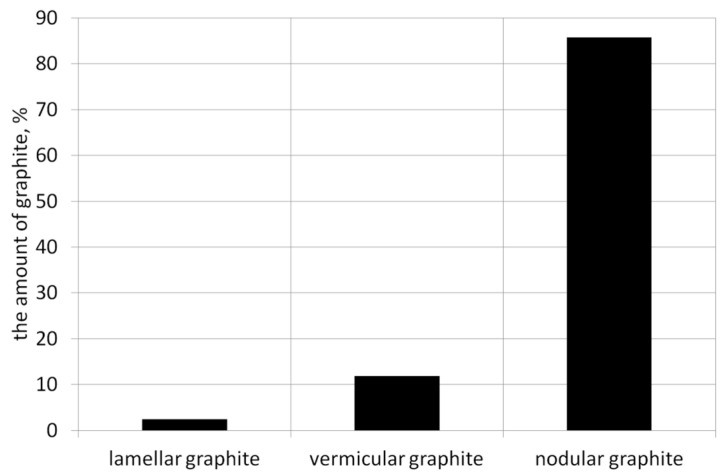
The share of individual types of graphite among all precipitates.

**Figure 9:**
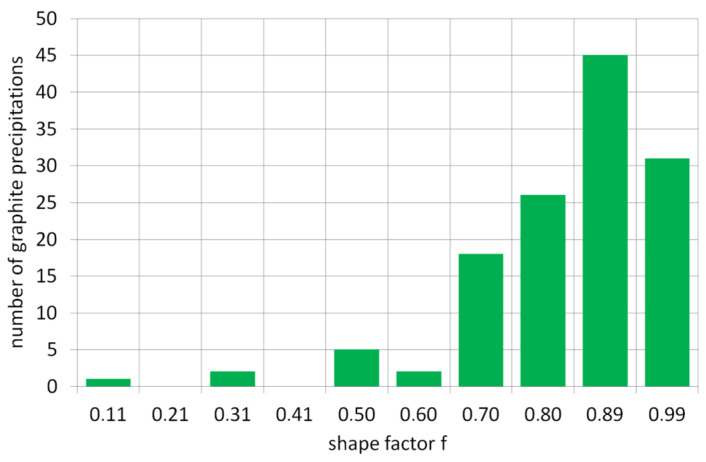
Number of graphite precipitates as a function of the aspect ratio.

Based on the calculation results summarized in Table 5, the average graphite shape indicator for this example is f = 0.78, which classifies the precipitates as spheroidal graphite. Since the percentage of graphite precipitates meeting the spheroidal shape criterion is 85.7%, the degree of spheroidization for this cast iron is N = 85.7%. Data from the selected example summarized in Table 5 are presented graphically in Figure 8 and Figure 9.

ImageJ, as demonstrated by the conducted analysis, offers a wide range of possibilities for a comprehensive quantitative description of graphite precipitates, even when the degree of shape heterogeneity of the graphite precipitates is significant. It allows for the determination of graphite precipitate indices such as:

- the average (weighted average) shape index of graphite “f_K_”
- the percentage of three graphite forms (Figure 8): flake, vermicular, and spherical.
- the distribution of graphite precipitate amounts by the “f” index value (Figure 9).
- the degree of spheroidization of cast iron “N,” defined as the percentage of graphite precipitates with a shape close to a sphere (f > 0.65).

The described methodology and procedure for determining the average shape indicator of graphite precipitates in Vari Morph cast iron have been used in all studies conducted to date in this field [1,2,3,4,5,6,7,8,9,10,11]. It can be assumed that thanks to the application of ImageJ and its adaptation to the description, the image of the structure of graphite precipitates is multi-parameter and sufficient for the needs of scientific analyses as well as practical applications in the study of cast iron grades with a complex structure in terms of the graphite shape, including Vari Morph cast iron.

## 4. Vari Morph (VM) Cast Iron Production Technologies

The research results presented in this paper represent a synthesis of many years of work by the authors. The first work on developing the Vari Morph cast iron production technology was published in 2018 [1]. The research demonstrated that VM cast iron can be produced using several methods, similar to the production of vermicular or ductile iron [8]. The technologies for producing mixed graphite cast iron are similar to the technologies for vermicular/ductile iron. Based on the analysis of the preliminary research results, the VM cast iron production method was selected, which involves secondary furnace treatment with low-magnesium alloys (master alloys), FeSiMg. This is a variant of producing cast iron that initially contains a limited sulfur content (S < 0.01%) by introducing a strictly controlled amount of magnesium (Mg < 0.03%), shown in Figure 3. The introduction of a controlled amount of Mg was accomplished in three ways (three methods):

(a) PE rod method—using a flexible hose containing low-magnesium FeSiMg mortar introduced into a ladle with a lid.
(b) Using low-magnesium mortars dosed to the bottom of tightly closed ladles (slim ladles—Tundish method).
(c) Inmold technology, placing low-magnesium mortars in reaction chambers constructed in a casting mold.

### 4.1. Controlled Magnesium Content Method

Pure magnesium or a magnesium mortar is added to the molten metal in an amount smaller than necessary to obtain nodular graphite. In vermicular cast iron, the final Mg content ranges from 0.01 to 0.03%, shown in Figure 3. After adding magnesium, modification is performed, usually with ferrosilicon-based modifiers, as in the production of ductile iron. Obtaining vermicular cast iron is difficult in industrial practice because the range of magnesium content in the cast iron at which graphite crystallizes in the vermicular form is very narrow. To produce VM cast iron with flake + vermicular graphite, the magnesium content must be maintained below 0.02%. To obtain VM cast iron with vermicular + nodular graphite, the Mg content should be maintained within the range of Mg = 0.02–0.03%. The indicated Mg contents apply to cast iron with a low sulfur content (S < 0.01%), and no magnesium is used for desulfurization. Excess Mg (Mg > 0.03%) will result in the formation of nodular graphite, while too little Mg will result in flake graphite. Such cast iron is also more susceptible to vermicularization failure than cerium-treated cast iron.

### 4.2. Proprietary Technologies to Produce Vari Morph Cast Iron

The research was initially conducted in the experimental foundry of the Faculty of Foundry Engineering at the AGH University of Science and Technology in Cracow, and then in a selected industrial foundry. The controlled Mg content method was chosen, and the spheroidization process was conducted using a ladle with a tight lid (Tundish technology) and the flexible conduit (PE) method. Inmold spheroidization was also performed under industrial conditions. Cast iron with a mixed graphite form (Vari Morph) is obtained using this technology by introducing a reduced amount of Mg compared to the amount in ductile iron. Therefore, the secondary metallurgy process, introducing Mg in a strictly controlled amount, can still be referred to as the spheroidization process of cast iron. This term is used throughout this paper. In the case of VM cast iron, spheroidization is incomplete, and the form of graphite precipitates varies widely across the three basic fractions in which it occurs in cast iron: flake (L), vermicular (V), and nodular (S).

**Figure 10:**
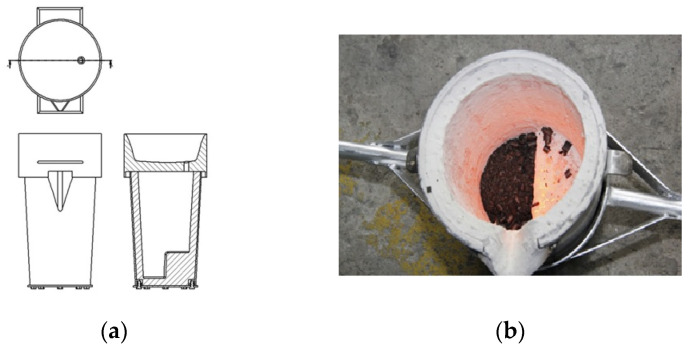
Slim, tightly covered ladle (Tundish): (**a**) sketch of the ladle: (**b**) FeSiMg mortar in the pocket of the heated Tundish ladle.

**Figure 11:**
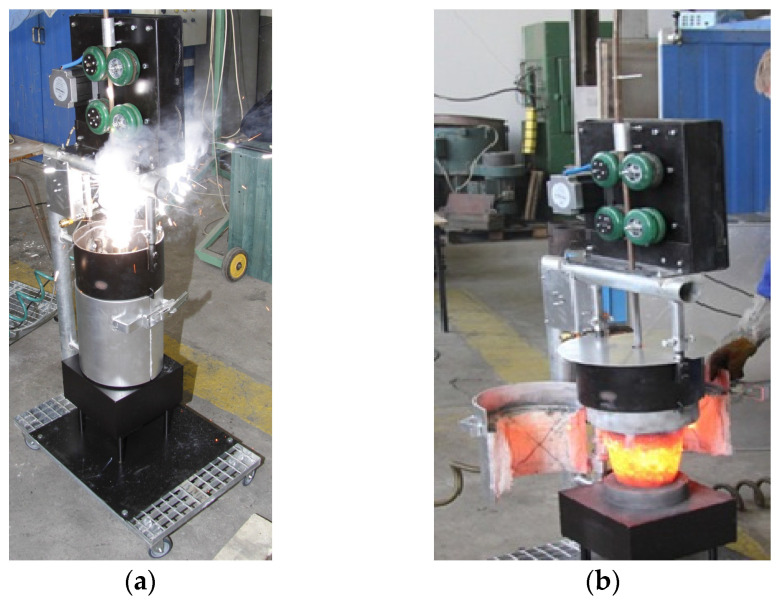
Experimental stand for spheroidization using the PE method (**a**) inserting the PE rod into the cast iron in the crucible with a cover. (**b**) metal in the crucible after spheroidization.

**Figure 12:**
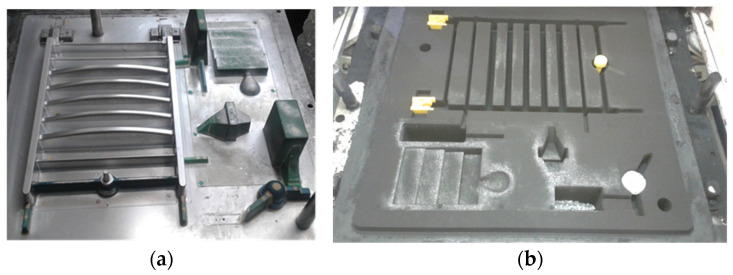
Test technology of Vari Morph cast iron casting in the Inmold process on the FBO line: (**a**) lower pattern plate, (**b**) lower mold before assembly and pouring.

**Figure 13:**
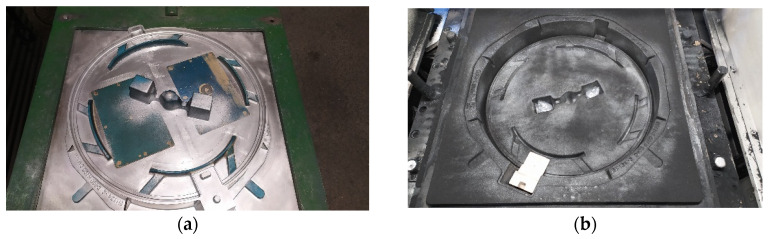
Pattern plate and sand mold of the road manhole body made of Vari Morph cast iron using Inmold technology on the FBO line; (**a**) pattern plate, (**b**) lower sand mold.

Figure 10 and Figure 11 show the experimental foundry layout, while Figure 12 and Figure 13 show the industrial layout. Figure 10a shows a sketch of the experimental Tundish ladle, and Figure 10b shows the heated vat with magnesium solution.

In the second solution of the Vari Morph cast iron production technology, spheroidization of cast iron was performed using the PE (flexible core wire) method. The experimental setup is shown in Figure 11. The cast iron spheroidization process involves introducing a steel tube (thin-walled pipe) filled with FeSiMg mortar, typically containing 16–23% magnesium, into the liquid metal. The process was carried out with full control of the rod immersion rate and the amount of mortar introduced, or more precisely, the amount of Mg introduced into the cast iron. The production of Vari Morph cast iron requires strict control of the amount of Mg introduced into the cast iron.

Production trials of Vari Morph cast iron were also conducted under industrial conditions as part of NCBiR projects [8]. Test ingots and test castings were made in molds with a horizontal surface on an automatic FBO molding line (Figure 12). The production of a drainage grate for street sewage was tested. Tests of the casting before its release for serial production include a test of the structural strength of the casting, which is determined by the ultimate strength of the material itself (R_m_, R_02_) and the casting design. It can be produced from various grades of cast iron, including Vari Morph cast iron with a graphite structure: vertical + spheroidal. The technology was evaluated on ingots/samples visible on the pattern plate (Figure 13a) and on the mold image shown in Figure 13b. The amount of magnesium introduced into the cast iron to obtain Vari Morph cast iron was controlled by selecting/calculating the amount of FeSiMg6 mortar introduced into the reaction chamber (Figure 13b). Inmold technology is the most stable and repeatable technology, in which the degree of magnesium assimilation is the highest, as is the quality of the cast iron in the casting. The quality of the cast iron results from the short time between the process of introducing magnesium into the metal and the solidification of the cast iron.

Research conducted in both the experimental foundry and industrial settings aimed to assess the feasibility of producing Vari Morph cast iron in a repeatable manner, with the intended structure of graphite precipitates, as well as to assess the possibility of creating selected physical, mechanical, and functional characteristics and properties. Comparison of three cast iron spheroidization methods allowed us to verify their effectiveness in introducing magnesium from FeSiMg master alloys into cast iron. This indicator is called the Mg assimilation rate and is a measure of the effectiveness of each method. The highest Mg assimilation efficiency is achieved with the Inmold technology (65–75%), slightly lower with the Tundish ladle spheroidization technology (55–60%), and the lowest with the PE flexible hose technology (30–35%). The Mg assimilation rate in each case and technology depends on a number of factors, including the cast iron temperature, sulfur (S) content, and oxygen (O_2_) content. However, the ranking of these technologies remains as indicated above.

This paper summarizes the results of research on unalloyed cast iron with a chemical composition close to the eutectic value (S_c_~1.0; CE~4.30) and reduced manganese content (Mn < 0.25). In most cases, the cast iron had a ferritic matrix, which allowed for the assessment of the effect on physical and mechanical properties of the graphite precipitate form, rather than the matrix type. The results of this work allowed for the determination of a number of empirical relationships between the graphite shape index and selected properties.

The group of cast iron grades whose chemical composition oscillated near the eutectic value was subject to investigation (C = 3.3–3.6%, Si = 2.6–2.95%, Mn < 0.25; P < 0.02%; S < 0.01%; Mg = 0.005–0.040%).

## 5. Research Results

### 5.1. Preparation of Research Material

Research on Vari Morph cast iron, in the first period, aimed to determine empirical relationships between the graphite shape, described by the shape indicator f_K_, and selected basic physical, mechanical, and functional properties [1,2,3,4,5,6]. In the second period, using and supplementing the earlier research, the aim was to determine similar relationships, but not so much as a function of the graphite shape indicator f_K_, but as a function of the basic parameter of ultrasonic wave propagation, i.e., its velocity. The relationship between the shape of graphite precipitates and wave velocity is generally known from many studies: the more compact the shape of the precipitates, the higher the velocity, which for the entire range of graphite shape changes ranges from approximately 3700 to 5900 m/s. Wave velocity is clearly less dependent on the metallographic matrix: pearlitic or ferritic. For the same form and amount of graphite, differences in wave velocity are within several hundred meters (200–400 m/s) [9,10].

The research presented below involved ferritic cast iron with a composition of approximately eutectic CE~4.26. Table 6 provides the chemical composition of cast iron from several dozen melts carried out in experimental foundries and industrial foundries. The ferritic matrix was obtained by limiting the content of elements that promote the formation of a pearlitic structure, and the basic composition was limited to Mn content < 0.10%.

Cast iron was prepared in induction furnaces from a charge consisting of blast furnace pig iron and cast iron scrap in varying proportions, depending on the actual composition of the charge materials. Furthermore, carburizer and ferrosilicon were added to the metal bath to regulate the composition of the starting cast iron. In the final melting phase and after the spheroidization process, a modification and secondary modification process were performed to refine the grains. In the final melting phase, the metal was briefly superheated to 1500–1520 °C to achieve degassing and flush out impurities. The pouring temperature ranged from 1420–1450 °C.

The test ingots themselves, in accordance with the standards in force in this respect, are type Y2 ingots. The ingots were cast from furan resin-based sands. During each test run, samples were also collected for spectrometric analysis. In all tests, the cast iron composition was carefully selected (Table 6) to allow for comparison of the tested material regardless of the casting location and spheroidization method. Among several methods for producing cast iron with controlled graphite content, the method of controlled residual Mg content in cast iron was selected for this study. To achieve the full spectrum of graphite precipitation forms, the Mg content varied (was controlled) from 0.005% to over 0.065%.

### 5.2. Determination of Empirical Dependencies

**Figure 14:**
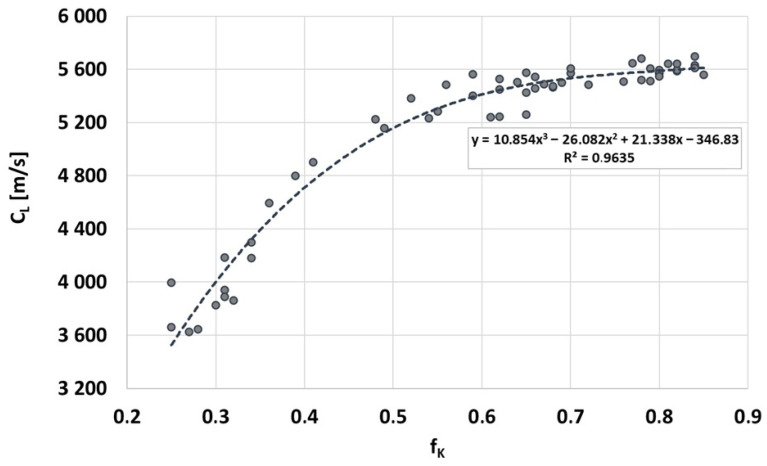
Relationship between the shape index of graphite precipitates f_K_ and the ultrasonic wave velocity in ferritic cast iron Vary Morph.

As mentioned earlier, the aim of the research was to determine the empirical relationship between ultrasonic wave velocity in Vari Morph cast iron and its physical, mechanical, and functional properties. The goal is to enable future practice to predict selected properties of Vari Morph cast iron based on non-destructive ultrasonic testing of castings. The ultrasonic testing described below was performed using Unipan CT-3 ultrasonic material testers with 2.0 MHz transducers. Wave velocity was assessed on a Y2 sample ingot, from which test samples were subsequently prepared. Figure 14 shows the key relationship obtained in the research: the relationship between the graphite shape index (f_K_) and the velocity of the longitudinal ultrasonic wave (C_L_). These two quantities, closely related to the shape of graphite precipitates, are well correlated. Wave velocity follows the spatial structure of the graphite precipitates, while the shape indicator is described by a flat image visible on the surface of the metallographic microsection. The more compact the graphite, the less dispersion of the propagating ultrasonic wave, resulting in its higher velocity. The greatest changes in wave velocity are observed in cast iron with a spatially expanded form, when the shape factor f_K_ reaches values below 0.50–0.60.

**Figure 15:**
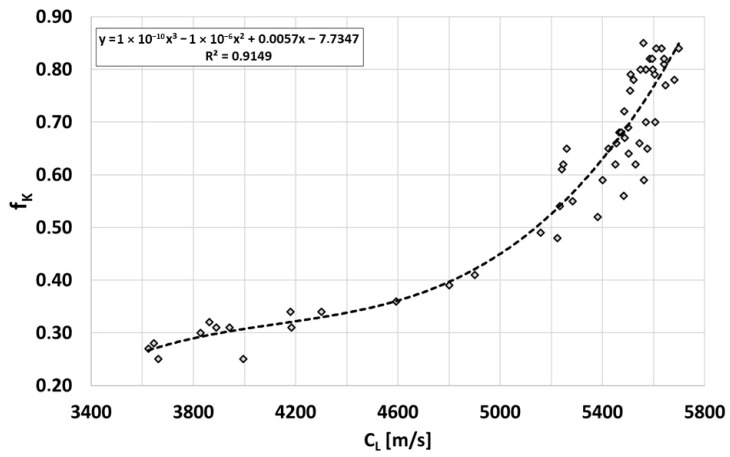
Relationship between the ultrasonic wave velocity and the shape indicator of graphite precipitates f_K_ in ferritic cast iron Vary Morph.

If the quantity measured in the sample/casting is the wave velocity, then on its basis and using the relationship in Figure 14, the value of the graphite shape indicator can be assessed, as shown in Figure 15. Measuring the ultrasonic wave velocity in a sample/casting made of Vari Morph cast iron, thanks to the connection with the graphite shape indicator f_K_, allows for indirect determination of all physical and mechanical properties of cast iron that depend on this particular f_K_ indicator. These properties and their dependencies on the f_K_ indicator value are described in [5,6].

**Figure 16:**
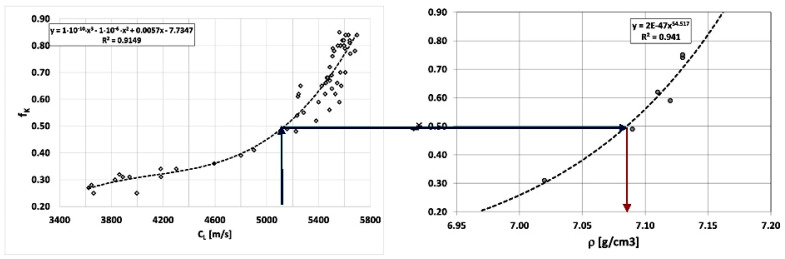
Interdependence of wave propagation velocity, averaged graphite shape index and specific density of ferritic Vari Morph cast iron.

When only the form of graphite changes in cast iron and the matrix is ferritic, the relationship between wave propagation velocity, the f_K_ indicator, and specific density can be determined, as shown in Figure 16. Density depends on the form of graphite, but determining an averaged shape indicator is difficult. It is easier to determine the velocity of the ultrasonic wave. Therefore, the authors searched for relationships between wave velocity and a whole group of physical and mechanical properties, knowing that in reality, they depend on the form of graphite precipitates (f_K_), as shown in Figure 16.

**Figure 17:**
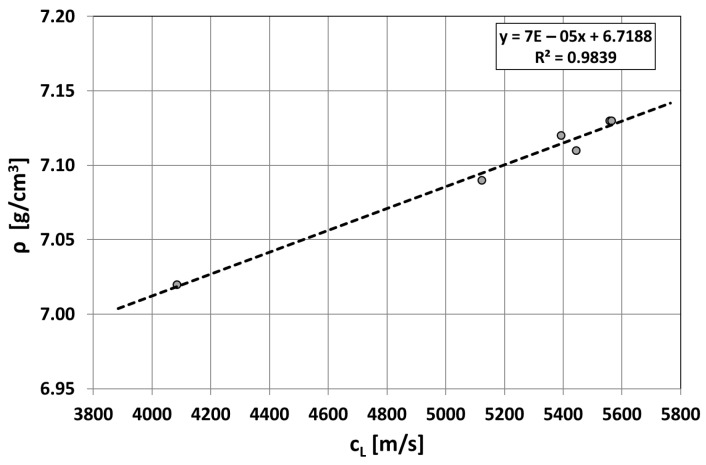
The relationship between the ultrasonic wave propagation velocity and the specific density of ferritic cast iron Vari Morph.

Further descriptions of the test results will present direct relationships between wave velocity in Vari Morph cast iron and the selected feature/property of the cast iron. The density of the cast iron was determined gravimetrically by measuring the sample weight in air and ethyl alcohol [5,6]. In the case of density tests, the obtained relationship is as shown in Figure 17. Within the tested range of density variation, which depends on the amount of graphite, the relationship ρ = f(C_L_) is linear. The more compact the graphite form and the associated higher wave velocity, the higher the density.

**Figure 18:**
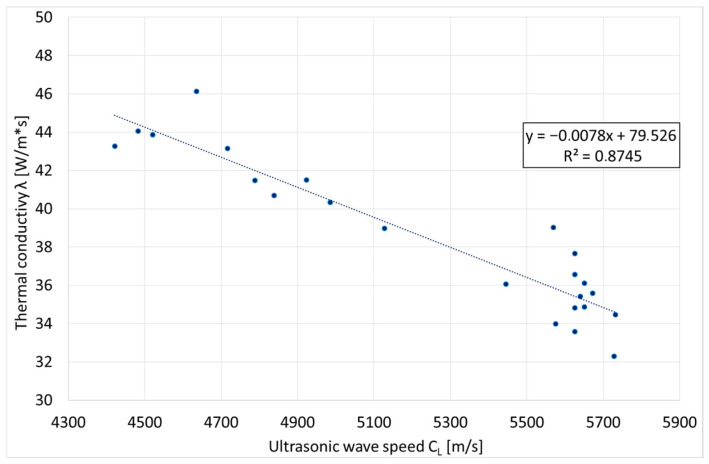
Relationship between ultrasonic wave propagation velocity (C_L_) and thermal conductivity of ferritic Vari Morph cast iron [4].

An important property and characteristic of Vari Morph cast iron is thermal conductivity (λ), which can vary widely with the shape of the graphite precipitates (f_K_). Many structural elements or structures themselves require thermal conductivity of a specific value, either smaller or larger. This is the case for vehicle brake discs, combustion engine housings, manifolds, and metallurgical equipment such as slag ladles and steel ingot molds, for which cast iron should be characterized by high thermal conductivity. The authors conducted research in this area, seeking an easy method for non-destructively determining the thermal conductivity of Vari Morph cast iron not only in test ingots but also in finished products/castings. The results of this work and the research methodology have been described in previous publications. The determined relationship of thermal conductivity (λ) as a function of longitudinal wave propagation velocity (C_L_) is presented in Figure 18.

Within the tested range of variability of Vari Morph cast iron, it is described by a linear equation. The expanded form of graphite promotes good thermal conductivity. For this reason, brake discs are manufactured from gray cast iron with a flake-like graphite form. Measuring thermal conductivity directly in castings has a significant advantage: the obtained results are averaged values. The metallographic structure on the cross-section of the wall of cast iron castings can vary widely. Averaging the conductivity values resulting from the measurement technique provides an image of the ability of heat to flow through the entire casting wall. This assesses the heat transfer capacity of the object/casting. In conventional thermal conductivity testing methods, thermal conductivity is most often performed on relatively small or very small samples (laser thermal diffusivity testing method). Translating from such a determined thermal conductivity to assessing the heat transfer capacity of a large object/casting is extremely difficult and often fraught with error.

**Figure 19:**
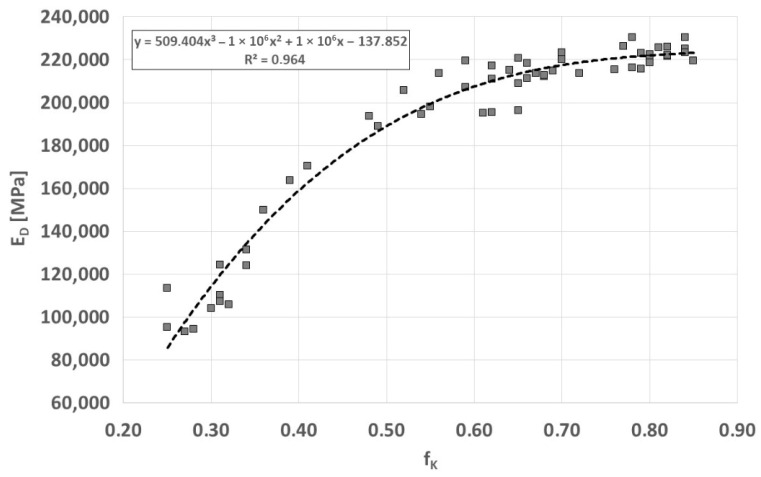
Relationship between the averaged shape index f_K_ of graphite and the dynamic modulus of elasticity of ferritic cast iron Vari Morph.

The dynamic modulus of elasticity E_D_, determined on the basis of a simplified equation using the measurement of wave velocity and specific density (E_D_ = ρ·(C_L_)^2^) in Vari Morph cast iron varies within wide limits, along with the change in the average graphite shape index f_K_, shown in Figure 19. The main changes in the modulus of elasticity are observed in cast iron with the shape index of graphite precipitates f_k_ < 0.6.

**Figure 20:**
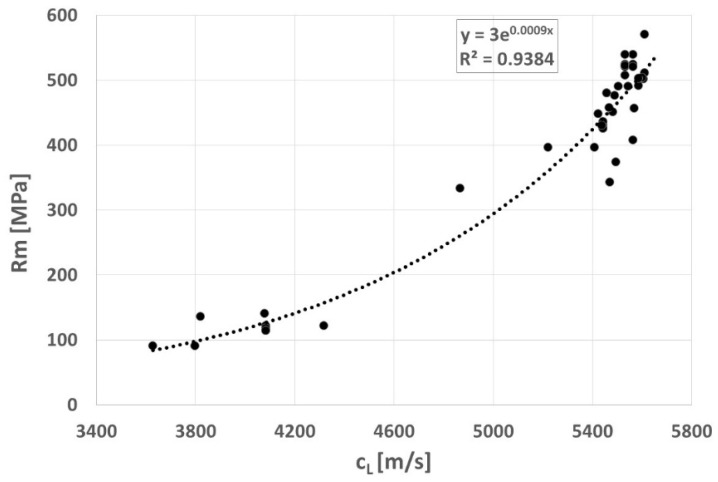
Relationship between ultrasonic wave velocity (C_L_) and strength R_m_ of ferritic cast iron Vari Morph.

**Figure 21:**
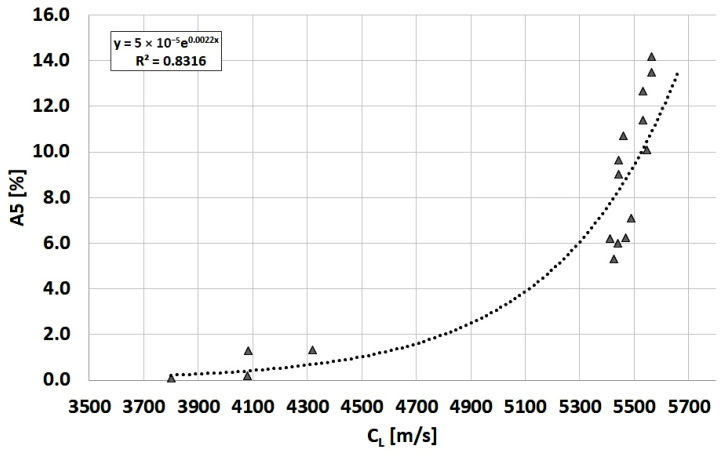
Relationship between ultrasonic wave velocity (C_L_) and A5 elongation of ferritic Vari Morph cast iron.

The form of graphite precipitates significantly affects both the strength (R_m_) and ductility of cast iron (A5). The influence of the graphite shape index f_K_ on these properties in Vari Morph cast iron was described by the authors in previous works. Because graphite shape indexes and ultrasonic wave velocity are well correlated, the authors determined the following relationships for Vari Morph cast iron: R_m_ = f(C_L_) and A5 = f(C_L_), shown in Figure 20 and Figure 21. These relationships apply to cast iron with a ferritic matrix. The ductility of cast iron measured by its elongation in a tensile test is characterized by a greater scatter of results and a lower correlation coefficient.

**Figure 22:**
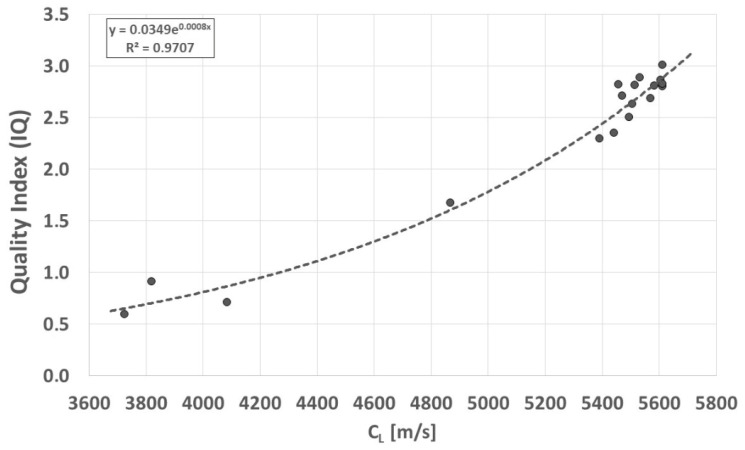
Relationship between ultrasonic wave velocity (C_L_) and iron quality index (IQ) of ferritic Vari Morph cast iron.

The IQ (Index Quality), defined for cast iron as the ratio of strength to hardness (IQ = R_m_/HB), is generally a measure of cast iron quality. In the case of VM cast iron, it specifically describes its quality. Changes in form have virtually no effect on the HB of the cast iron, which is determined by the type of matrix. The IQ value of cast iron increases in ferritic VM cast iron in proportion to the increase in the graphite aspect ratio [2,5,6]. In ultrasonic inspection of Vari Morph cast iron, with increasing speed, the QI value increases progressively, shown in Figure 22. The definition of the IQ index suggests that an increase in its value indicates a greater increase in strength than in hardness, which is caused by changes in the metallographic structure. This tendency to increase R_m_ while maintaining a constant HB is particularly advantageous in the case of subtractive/finishing machining of cast iron castings. Ferritic matrix castings, despite the high strength obtained by increasing the graphite aspect ratio “f_K_,” can be very easily machined using CNC machine tools.

**Figure 23:**
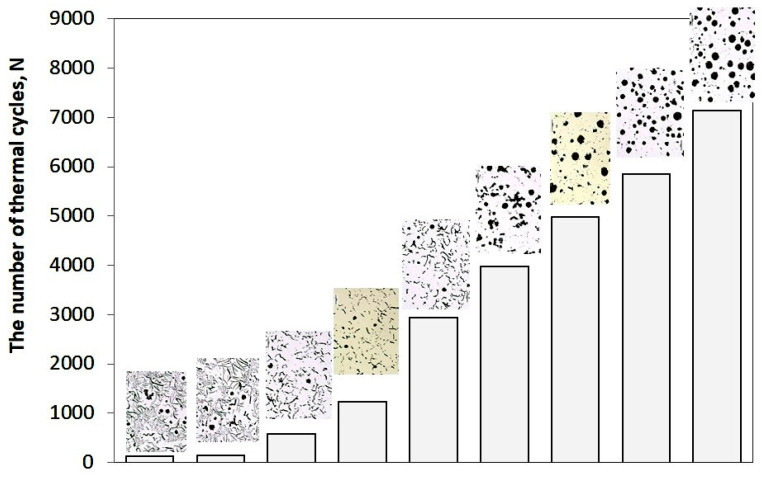
The influence of the form of graphite precipitations on the thermal fatigue resistance of cast iron determined using the L.F. Coffin method, T_min_ = 200 °C; T_max_ = 650 °C.

**Figure 24:**
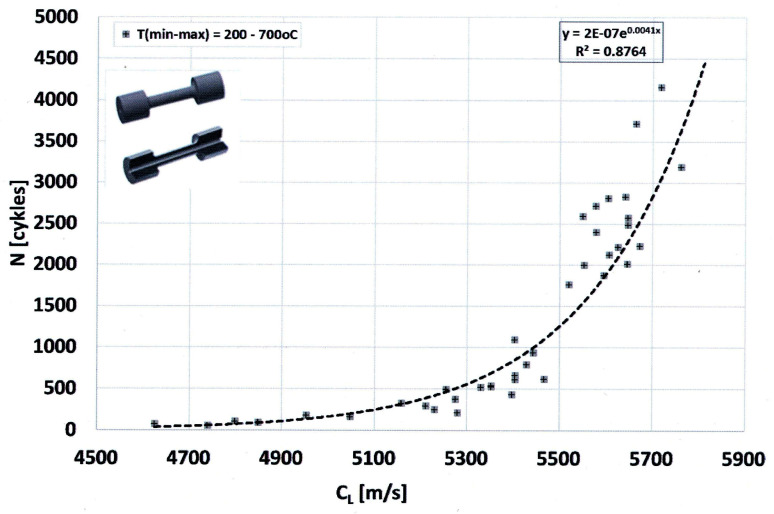
Relationship between ultrasonic wave velocity (C_L_) and thermal fatigue resistance of ferritic Vari Morph cast iron. L.F. Coffin method, test parameters; T_min_ = 200 °C; T_max_ = 700 °C.

Thermal fatigue resistance of metals is one of the most complex functional properties of metals, generally dependent on the metallographic structure of the alloy and the operating conditions of the component. The complexity of this property stems from the fact that it is linked to such physical and mechanical properties as thermal conductivity and expansion, elasticity, plasticity, and strength (including at elevated temperatures). Many methods exist for testing thermal fatigue resistance. This paper presents the results of thermal fatigue resistance tests obtained using resistance heating of rod-shaped samples (L.F. Coffin’s method [10,11]). Vari Morph cast iron with a ferritic matrix was tested. An empirical relationship was determined between the thermal fatigue resistance of cast iron (the number of thermal cycles N to sample fracture) and the graphite shape indicator (f_K_): N = f(f_K_), as well as a relationship in which the cast iron structure was assessed using ultrasonic testing: N = f(C_L_). Both relationships are power-law functions—as the graphite shape indicator increases, the thermal fatigue resistance of cast iron increases. Similarly, as the wave velocity in the tested samples increases, their thermal fatigue resistance increases. The influence of the shape of graphite precipitates on thermal fatigue resistance is shown in Figure 23. The tests were conducted in the temperature range of the 200–650 °C cycle. Quantitatively, and at a slightly higher maximum temperature (200–700 °C range), the relationship shown in Figure 24 was obtained for ferritic cast iron. Therefore, based on the measurement of ultrasonic wave velocity, the behavior of cast iron (a part made of cast iron) under cyclic heating conditions can be predicted. Naturally, this is an estimate, as the actual resistance and durability of components operating under cyclic heating conditions depends on several other factors, including the degree of free thermal expansion of the heated component [12].

**Figure 25:**
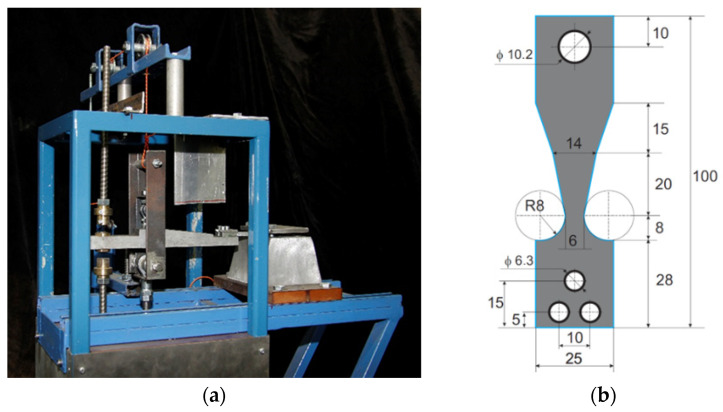
Appearance: (**a**) low-cycle fatigue test stand, (**b**) sample subjected to bending.

Low-cycle mechanical fatigue, the material’s resistance to this type of loading in the case of cast iron, is primarily dependent on the form of graphite precipitates, the graphite shape indicator f_K_. Mechanical fatigue tests were conducted under classical bending conditions on samples clamped unilaterally. Bilateral symmetrical bending was performed at a frequency of 4 Hz and an amplitude of 2.0 mm. The tests were conducted on a prototype test stand shown in Figure 25a. The samples were designed so that the stress value along the entire length of the sample was approximately constant during bending, as shown in Figure 25b.

**Figure 26:**
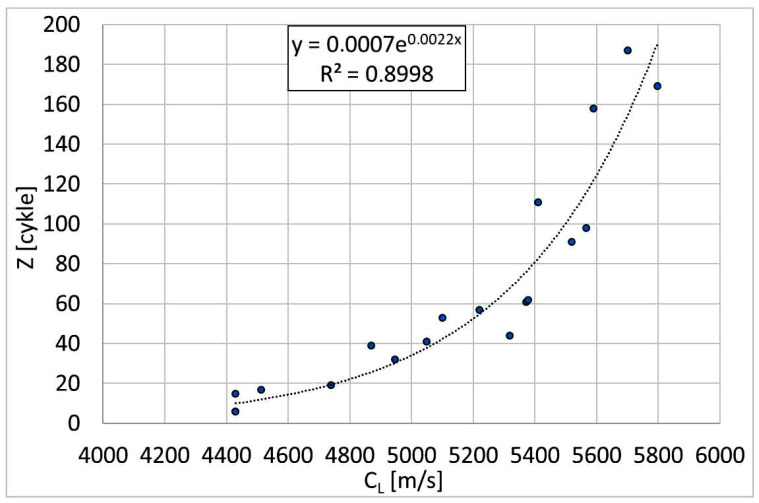
Relationship between ultrasonic wave velocity (C_L_) and low-cycle mechanical fatigue (bending) resistance of ferritic Vari Morph cast iron.

The aim of this study was to determine the relationship between the form of graphite precipitates, controlled indirectly by measuring the ultrasonic wave velocity (C_L_), and the number of bending cycles (Z_c_) that the sample endures before fracture. Figure 26 shows the obtained empirical relationship Z_c_ = f(C_L_). This relationship is of a power-law nature, confirming the significant influence of the graphite form on the low-cycle fatigue resistance of VM cast iron.

**Figure 27:**
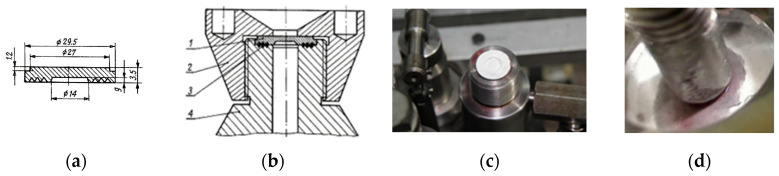
Sketches and photos of cast iron tightness sample and laboratory stand. (**a**) Cast iron leak test sample; (**b**) Sketch of the device head: 1-sample, 2-nut, 3-gasket, 4-head; (**c**) Placing the sample in the measuring head; (**d**) Medium permeation through the sample.

**Figure 28:**
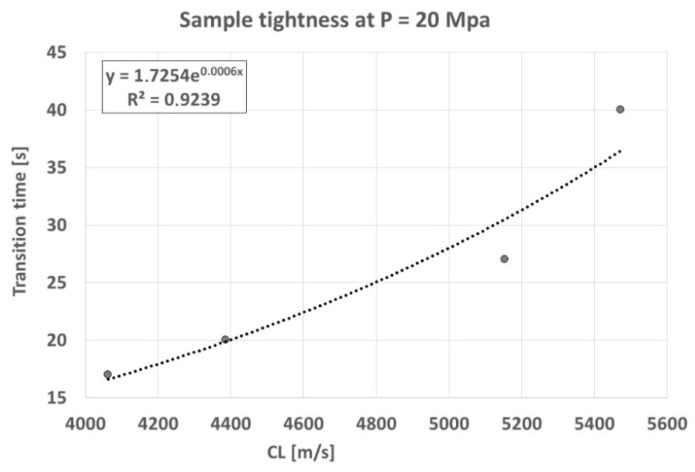
Relationship between ultrasonic wave velocity (C_L_) and tightness of ferritic cast iron Vari Morph.

Tightness is the ability of a wall to resist liquids and gases that exert a specific pressure on it and attempt to penetrate it. This material characteristic, in the case of cast iron castings, is of particular importance in many manufacturing sectors. This is primarily the automotive industry, the construction of electric motors, compressors, water and gas fittings, boilers, and radiators, where tightness is the primary criterion for assessing casting quality. In research on the effect of graphite precipitate shape on the tightness of “Vari Morph” cast iron, with variable graphite form, an empirical relationship was sought between the cast iron’s tightness index (e.g., time to penetration at constant pressure) as a function of the average shape index “f_K_.” The authors described the results of these studies in [12]. The tests were conducted on samples made from test ingots; the dimensions of the sample and test stand construction are given in Figure 27. A pressure of P = 20 MPa was applied to the sample in a measuring head filled with measuring liquid (technical kerosene), and the time required for the medium to penetrate the sample surface was determined. Vari Morph cast iron with various f_K_ index values was tested. The tightness measurement results as a function of the ultrasonic wave velocity characteristic for each sample are presented in Figure 28. Ultrasonic wave velocity in VM cast iron can be a good indicator of the predicted tightness of cast iron castings. Its measurement on finished iron products/castings can be helpful in assessing the suitability of these products for high-pressure operation—structures/die-castings. The higher the C_L_ velocity, the more compact the graphite (the higher the f_K_ index value) and the greater the tightness of the casting.

Summary: Work on the technology of production and application of cast iron with graphite in vermicular and compact form was carried out by many authors and is described, among others, in works [18,19,20,21,22,23]. The presented results of multidirectional research constitute a synthesis of several years of work by the authors on cast iron with a variable but intentionally controlled form of graphite precipitations in cast iron with a ferritic matrix. They complement the results described in [1,2,3,4,5,6,7,12]. This time, attention was focused on finding the relationship between ultrasonic wave propagation in Vari Morpf cast iron and a group of physical, mechanical, and functional properties. The wave velocity, as shown in the work, is well correlated with the form of graphite precipitations, which is a leading factor for almost all (except HB) properties of cast iron. The following empirical relationships were determined: C_L_ = f(f_K_), density as a function of ρ = f(C_L_); modulus of elasticity E_D_ = f(C_L_); thermal conductivity λ = f(C_L_); tensile strength R_m_ = f(C_L_); elongation A5 = f(C_L_); cast iron quality index IQ = f(C_L_); thermal fatigue resistance N = f(C_L_); tightness of cast iron H = f(C_L_). The described relationships are relatively well correlated and can be used in practice to predict/forecast the properties in the walls of castings made of ferritic Vari Morph cast iron as well as to predict the behavior of structures/castings under specific operating conditions, e.g., when tightness of castings is required or when castings are subject to thermal fatigue.

## 6. Examples of Mixed Graphite Iron Castings (Vari Morph)

In industrial applications, mixed graphite castings are very common, primarily in the production of vermicular or ductile iron. They can be incorporated into the casting structure, with different metal crystals in the wall. This occurs due to separate effects that can be caused by spheroidization and pouring momentum through the molds. Naturally, the diversified graphite structure applies to cast iron grades that are subject to an independent spheroidal casting method in the liquid state. However, the ruling notice should not be considered a classification as Vari Morph cast iron. VM is a grade of cast iron with mixed graphite, whose VM structure was intentionally created using the spheroidization process. The characteristics of cast iron obtained through the use of non-standard cast iron grades are available from the perspective of their practical application. Thanks to the new configuration of the graphite precipitates, it is possible to control properties such as resistance to temperature changes and thermal shocks, tightness of cast iron, extended range of quality IQ and higher efficiency at a comparable or additional R_m_ values.

**Figure 29:**
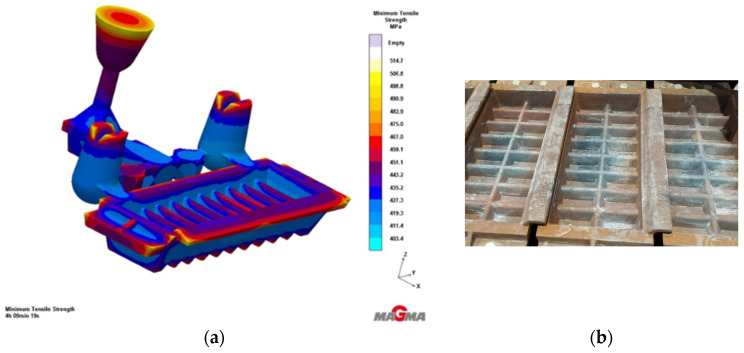
Ingot molds for pouring Al-Si alloys made of vermicular cast iron and Vari Morpf—Inmold technology; (**a**) pouring and solidification simulation in Magma Soft 6.1-Cast Iron, (**b**) castings on the pouring line.

A good example of the use of VM cast iron is all kinds of ingot molds, whose properties are a compromise between good thermal conductivity (Figure 18) and high resistance to thermal fatigue (Figure 24). Ductile iron (low thermal conductivity) does not meet these expectations, while vermicular cast iron (moderate resistance to thermal fatigue) is also not the best material to work in the conditions described. It would be advantageous to use VM cast iron with a graphite shape index f_K_ in the range of 0.60–0.75, which corresponds to the wave velocity range C_L_ = 5450–5550 m/s. An example of such a casting, an ingot mold for pouring aluminum, is shown in Figure 29 [21].

**Figure 30:**
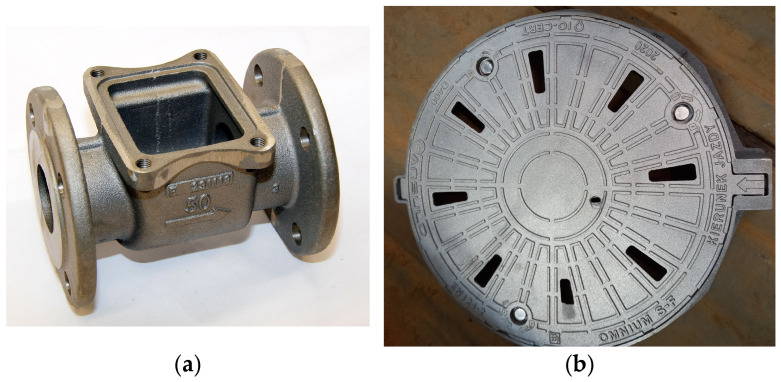
Examples of applications of Vari Morph cast iron: (**a**) for castings with increased tightness—gas meter valve body; (**b**) for castings with increased strength and the ability to dampen vibrations (silence operation)—D400 manhole cover; ductile iron cover + VM cast iron body.

The group of castings tested from Vari Morph cast iron within the project [8] includes gas meter valve bodies, which are expected to be highly tight (Figure 30a), and castings dedicated to road sewers—manhole covers and sewer grates (Figure 30b). Valve bodies are currently made of EN GJL—250 cast iron; replacing it with Vari Morph cast iron allows for reducing the wall thickness by at least 10% while maintaining the required tightness. In the case of manhole covers, to maximize the weight of the castings, the covers are made of ductile cast iron EN GJS 500-7. Channel bodies in which mainly compressive stresses occur during operation can be made of VM cast iron, which in the Inmold technology is easily achieved by reducing the amount of FeSiMg mortar dosed into the reaction chamber. VM cast iron dampens vibrations more effectively, which helps extinguish them after a vehicle wheel passes through the hatch. This arrangement is recommended for micro-environment-friendly operation of the body and cover assembly (quiet operation). In Reference [5], the authors provide further examples of the use of Vari Morph cast iron in various technical and industrial sectors.

## 7. Conclusions

Research studies were conducted as a continuation of publications [1,2,3,4,5,6,7,8,9,10,11,12] on Vari Morph cast iron. Confirmation of the possibility of producing cast iron with graphite shapes in many forms, called Vari Morph, in a targeted and controlled manner was obtained. It was also claimed that it could be produced using several methods in experimental foundries, as well as in industrial foundries. Inmold technology ensures the production of this cast iron. The combination of the instantaneous introduction of Mg-protected graphite into the liquid cast iron and the moment of solidification in the mold cavity allows for the prediction of the amount of Mg required for the cast iron. In the case of spheroidization technology, magnesium evaporates during the spheroidization process until the moment of the control method. Industrial trials have been thoroughly verified.

Vari Morph cast iron has a number of properties characterized by high IQ quality indicators. It fills the area of a specific discontinuity in the isolated graphite compared to the definition of standardized cast iron grades with compatible graphite: flake, vermicular, or spheroidal. VM cast iron is an attractive material for structures and castings requiring above-standard properties that cannot be reused when graphite is functional. As demonstrated in this work, it is suitable for structures operating under conditions of rapid temperature changes, leak-releasing emissions, easily machined castings, etc. Vari Morph cast iron is considered a future-proof application, filling a gap in the research offering cast iron grades.

Expanding on earlier work [1,2,3,4,5,6,7,8,9,10,11], the authors demonstrated that the properties of VM cast iron with a homogeneous ferritic matrix, previously described as a function of the graphite shape index, can be described by empirical relationships whose independent variable is the ultrasonic wave velocity. They therefore propose that the graphite form in VM cast iron be assessed and monitored non-destructively using ultrasonic techniques, commonly used in iron foundries. Ultrasonic testing of cast iron based on wave velocity is a very convenient inspection tool, previously used mainly for the inspection of ductile cast iron. It can be even more applicable to the study of the form of precipitates in VM graphite.

Within the entire range of ultrasonic wave velocity changes from gray cast iron with flake graphite to ductile cast iron with spherical graphite, wave velocity ranges characteristic for individual cast iron grades can be distinguished, as shown illustratively in Figure 3. And so, gray cast iron with a ferritic matrix—C_L_ < 4800–4900 m/s; VM cast iron with a mixture of flake and vermicular graphite C_L_ < 4800–5200 m/s; cast iron with vermicular graphite C_L_ < 5200–5350 m/s; VM cast iron with vermicular and nodular graphite: CL—5350–5500 m/s; ductile cast iron with nodular graphite; C_L_ > 5500 m/s. Naturally, these are approximate limits of the described ranges.

Investigations performed in this study allowed us to empirically determine dependencies existing between the ultrasonic wave velocity and the selected property, feature or functional property of VM cast iron. Like all empirical relationships, they are subject to some error, but they allow for a very quick and comprehensive assessment of cast iron with graphite, regardless of its form. Their use allows for the prediction of a whole group of properties and characteristics of cast iron in the finished product—a casting made from cast iron in which the matrix is purely ferritic or close to fully ferritic.

These are dependencies of:

- physical properties (graphite shape indicator f_K_, specific density, thermal conductivity, ultrasound wave speed): f_K_ = f(C_L_); ρ = f(C_L_); λ = f(C_L_),
- mechanical properties (strength (Rm), A5, coefficient of elasticity E_D_, quality index IQ): Rm = f(C_L_); A5 = f(C_L_), E_D_ = f(C_L_), IQ = f(C_L_),
- functional properties (heat fatigue resistance (N), low-cycle mechanical fatigue Z, cast iron tightness H): N = f(C_L_); Z_c_ = f(C_L_), Z = f(C_L_)

## Figures and Tables

**Table 1:** Examples of graphite precipitate morphology for different values of the shape indicator f_K_.

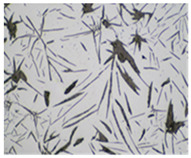 f_K_ = 0.28	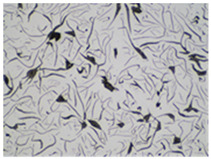 f_K_ = 0.29	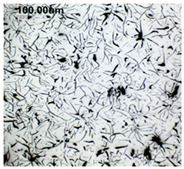 f_K_ = 0.31	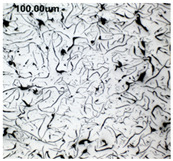 f_K_ = 0.32
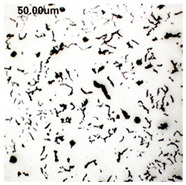 f_K_ = 0.42	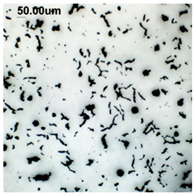 f_K_ = 0.50	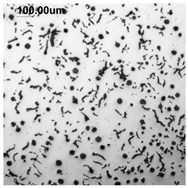 f_K_ = 0.59	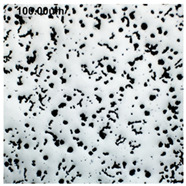 f_K_ = 0.62
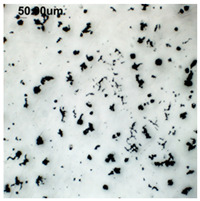 f_K_ = 0.63	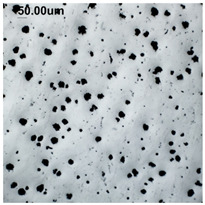 f_K_ = 0.72	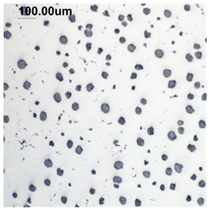 f_K_ = 0.78	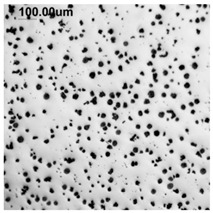 f_K_ = 0.82

**Table 2:** Graphite shape indicator values ξ, and “f” calculated according to various equations.

Graphite section	Calculation formula	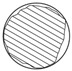	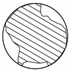	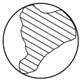	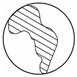	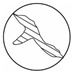
$\frac{Graphite\;surface\;area}{Circle\;surface\;area}\;\%$	$f=\frac{A_{graph.}}{A_{circle}}\cdot 100$	90.8	79.5	57.5	34.6	12.7
$\frac{Graphite\;surface\;area}{Circle\;surface\;area}$	$f=\frac{A_{graph}}{A_{circle}}$	0.91	0.80	0.58	0.35	0.13
Graphite surface areaGrapfit diameter (P)^2	$\xi =\frac{A_{graph.}}{P^{2}}$	0.080	0.070	0.060	0.045	0.030

**Table 3:** Spatial and flat images of graphite precipitation in three types of cast iron.

SEM Breakthrough Image	Image of a Metallographic Cross-Section
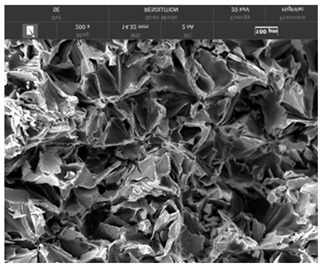 a/	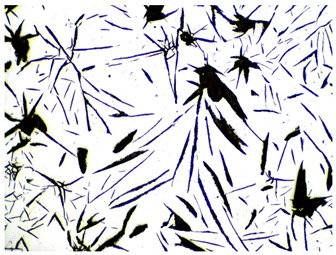 b/
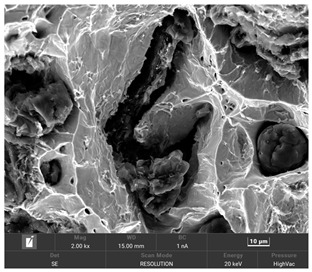 c/	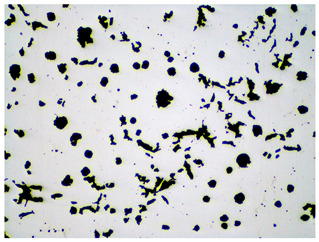 d/
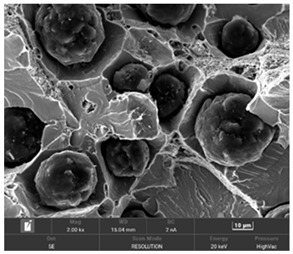 e/	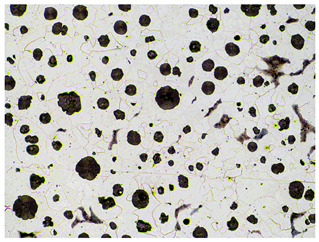 f/

**Table 4:** Selected parameters of graphite precipitation in VM cast iron.

Parameter	Value
Average number of precipitates per mm^2^	130
Total surface area occupied by graphite precipitates	10%
Average Feret diameter	0.03529440 mm
Average precipitate circumference	0.10759123 mm

**Table 5:** Analysis of the shape index “f” and calculation of the average value of the f_K_ index.

1	2	3	4	5
Graphite Type	Number of Precipitates	Percentage Share [%] (Degree of Spheroidization N)	the Average Value of the “f” Index in the Range	“f_K_” Index (Weighted Average)
flake	3	2.4	0.18	0.78
vermicular	15	11.9	0.56	
spheroidal	108	85.7	0.83	
**∑**	126	100%		

**Table 6:** Chemical composition of basic elements.

C [%]	Si [%]	Mn [%]	P [%]	S [%]	Mg [%]
3.3–3.6	2.6–2.95	<0.1	<0.02	<0.01	0.005–0.065

## Data Availability

The original contributions presented in this study are included in the article. Further inquiries can be directed to the corresponding author.
